# Pathways for maintenance of telomeres and common fragile sites during DNA replication stress

**DOI:** 10.1098/rsob.180018

**Published:** 2018-04-25

**Authors:** Özgün Özer, Ian D. Hickson

**Affiliations:** Center for Chromosome Stability and Center for Healthy Aging, Department of Cellular and Molecular Medicine, University of Copenhagen, Blegdamsvej 3B, 2200 Copenhagen N, Denmark

**Keywords:** alternative lengthening of telomeres, common fragile sites, RAD52, homologous recombination, cancer

## Abstract

Oncogene activation during tumour development leads to changes in the DNA replication programme that enhance DNA replication stress. Certain regions of the human genome, such as common fragile sites and telomeres, are particularly sensitive to DNA replication stress due to their inherently ‘difficult-to-replicate’ nature. Indeed, it appears that these regions sometimes fail to complete DNA replication within the period of interphase when cells are exposed to DNA replication stress. Under these conditions, cells use a salvage pathway, termed ‘mitotic DNA repair synthesis (MiDAS)’, to complete DNA synthesis in the early stages of mitosis. If MiDAS fails, the ensuing mitotic errors threaten genome integrity and cell viability. Recent studies have provided an insight into how MiDAS helps cells to counteract DNA replication stress. However, our understanding of the molecular mechanisms and regulation of MiDAS remain poorly defined. Here, we provide an overview of how DNA replication stress triggers MiDAS, with an emphasis on how common fragile sites and telomeres are maintained. Furthermore, we discuss how a better understanding of MiDAS might reveal novel strategies to target cancer cells that maintain viability in the face of chronic oncogene-induced DNA replication stress.

## 1. Introduction

Genome instability is a defining hallmark of cancer [1] and is thought to promote tumorigenesis in pre-cancerous lesions, as well as karyotypic diversity (and hence cellular heterogeneity) during cancer progression [2–4]. There are a number of hypotheses for why tumour cells exhibit intrinsic genomic instability. These can be broadly classified into two categories: those that posit a requirement for genomic instability in the tumorigenesis process, and those that propose instability is merely a by-product of other genetic changes that occur during tumorigenesis. With respect to the latter, it is clear that loss of tumour suppressor gene function often disrupts genome maintenance pathways. Genome instability in cancer is also induced by the activation of oncogenes. The ability of oncogenes to induce cell cycle entry and cell proliferation is well established. However, another consequence of oncogene activation, and the one of most relevance to the subject of this article, is the induction of chronic ‘DNA replication stress’. This term refers to any condition that leads to the slowing and/or stalling of replication forks. Chronic DNA replication stress has now been observed in a wide range of tumours and, as a consequence, has been proposed as an additional hallmark of cancer [5,6].

Activation of oncogenes in the early stages of tumorigenesis leads to activation of replication, double-strand break (DSB) formation, and a DNA damage response. In turn, the induction of a DNA damage response results in senescence or apoptosis in normal cells, which acts as a barrier to tumour formation. Cancer cells, on the other hand, prevent senescence or apoptosis by inactivation of tumour suppressor proteins, such as p53, and can thus tolerate much higher levels of chronic DNA replication stress [5,7,8]. Although oncogenes have been shown to increase replication origin firing and depletion of nucleotide pools, increasing evidence points towards replication–transcription collisions as the underlying cause of oncogene-induced DNA replication stress [9–13]. A recent genome-wide mapping study of the DNA replication and transcription sites provided further insight into the mechanism of transcription–replication conflicts induced by oncogenes [12]. Over-expression of oncogenes was shown to induce premature entry into S-phase from G1, and the activation of new replication origins located within highly transcribed regions. This origin activation within protein-coding genes led to replication fork collapse, DSB formation and chromosomal translocations [12,14,15].

Given the prevalence of DNA replication stress in tumorigenesis, it is imperative to understand the defence mechanisms that cancer cells use to tolerate this stress. This would afford us a potential opportunity to target a cancer cell-specific vulnerability. One such defence mechanism that was described recently in our laboratory is the activation of an atypical type of DNA synthesis that occurs in the early stages of mitosis [16]. This process, which we have termed MiDAS (for mitotic DNA synthesis), appears to be a form of homologous recombination-based DNA repair. MiDAS is more prevalent in aneuploid cancer cells (or otherwise transformed cells), where it counteracts DNA replication stress that arises at ‘difficult-to-replicate’ loci. In this article, we review the underlying mechanisms that are believed to prevent these loci from being duplicated in a timely manner. Furthermore, we discuss how MiDAS serves as a salvage pathway to ensure completion of genome-wide replication and hence prevent pathological chromosome mis-segregation events.

## 2. Difficult-to-replicate loci—or the ‘enemies within’ the genome

There are certain regions in the human genome that are inherently difficult to replicate. The best characterized examples are the ribosomal DNA (rDNA), chromosome fragile sites and telomeres [17]. These regions share at least some of the following features: they contain repetitive and/or G-rich sequences, which tend to form DNA secondary structures; they are associated with tightly bound protein complexes; they harbour unusually long genes or highly transcribed regions that increase the likelihood of collisions between the transcription and replication machineries; they are packaged into heterochromatin. Any or all of these features pose challenges to the replication machinery that might impede replication fork progression [18,19]. Moreover, these regions frequently give rise to ultra-fine anaphase bridges (UFBs), which connect the separating sister masses of DNA during the anaphase of mitosis. UFBs cannot be visualized using conventional DNA dyes, and are detectable by immuno-staining for certain proteins that coat the UFB, such as BLM and PICH [20]. In the following sections, we review the key similarities and differences between the DNA replication characteristics of these ‘difficult-to-replicate’ regions.

### 2.1. rDNA

The rDNA consists of tandem repeats of DNA units that encode the rRNA required for protein translation. The rDNA poses a challenge for the replication machinery because it is so highly transcribed, and hence DNA replication–transcription conflicts are inevitable. rDNA loci require specialized proteins/mechanisms to maintain the stability of each individual rDNA unit [21,22]. The high levels of transcription at the rDNA need to be coordinated with DNA replication in order to prevent the transcription and replication machineries from colliding. This process is orchestrated by a dedicated replication fork barrier positioned within each rDNA unit. Such barriers have been characterized extensively in yeast, but also have been shown to play an important role in human cells [17,23]. Despite the presence of the replication fork barrier, the rDNA array in yeast frequently segregates late in mitosis, probably due to the late completion of replication at that site. The rDNA is also prone to generate RNA : DNA hybrids (R-loops), in which the RNA transcript base pairs with the DNA template and displaces the complementary DNA strand. If not removed in a timely manner, R-loops can disrupt the function of underlying genes, and pathological R-loops are generally associated with loci where replication–transcription collisions are prevalent. As a consequence, the rDNA is a hotspot for transcription-driven mutagenesis/recombination [24–29].

### 2.2. Fragile sites

Fragile sites are regions of the genome that are prone to form visible gaps and breaks on metaphase chromosomes following perturbation of DNA synthesis. Fragile sites are categorized as being either ‘common’ or ‘rare’ according to their prevalence in the general population. Rare fragile sites are seen only in a small percentage of the population and are caused by pathological expansion of trinucleotide repeat sequences. Common fragile sites (CFSs), by contrast, exist in all individuals (reviewed in [30]). CFSs are frequently associated with the breakpoints of genomic rearrangements in cancer cells, as well as with micro-deletions and copy number variations (reviewed in [31,32]). Several mechanisms have been proposed to explain the sensitivity of CFSs to DNA replication stress, although the precise underlying cause of fragility may vary between different CFSs. However, CFSs are widely regarded as being the last regions of the human genome to be replicated [33]. CFSs tend to have an AT-rich sequence composition, which can lead to the formation of DNA secondary structures that can impede replisome movement. Coupled with their general lack of active/dormant replication origins, this might potentiate DNA replication stress during S-phase. However, perhaps the most striking feature of CFSs is their propensity to harbour large, actively transcribed genes that take at least one full cell cycle to transcribe. As a consequence of this, a collision between the replication and transcription machineries on the same template is inevitable. These collisions may generate DNA damage and/or lead to the formation of pathological R-loops [34–37].

The formation of breaks and gaps at CFSs on metaphase chromosomes is often referred to as fragile site ‘expression’ [32,38,39]. The differential cell/tissue specificity of CFS expression most likely reflects differences in the intrinsic transcription and replication profiles in the different cell types, but could also reflect an altered density of active replication origins (such as would be generated as a result of oncogene activation; see above) or chromatin structure (such as by histone hypo-acetylation) in different cell types [40–44]. The most common way to induce CFS expression in cultured cells is to expose them to the replicative DNA polymerase inhibitor aphidicolin [45]. Interestingly, this can induce micro-deletions at CFSs, a phenomenon similar to that seen in primary human tumours [46]. Activation of oncogenes, which occurs during cancer development, can also induce CFS expression [44], and recurrent deletions in cancer have been mapped to CFSs [44,47]. The most frequently expressed and best-characterized CFSs in the human genome are FRA3B and FRA16D, which harbour the tumour suppressor genes *FHIT* and *WWOX*, respectively [34,45,48,49].

CFSs are conserved through mammalian evolution, despite their propensity to induce genomic instability [30]. The reason for this high level of conservation is not known. Several explanations have been proposed. First, in addition to defined gene products, these loci encode crucial regulatory noncoding sequences, such as miRNAs [50]. Second, CFSs may act as a ‘sensor’ for alerting the cell to a failure to complete replication of the genome. If true, it seems likely that this function must be overwhelmed in situations where the cell encounters high levels of DNA replication stress, such as during oncogene-induced tumorigenesis [16,30]. Another speculative role for CFSs is that they serve to alert cells to the presence of invading organisms such as viruses that generate DNA replication stress as they seek to subvert the DNA synthesis machinery of the host in order to propagate themselves.

Several DNA repair/DNA damage response proteins have been implicated in CFS maintenance, including the Fanconi anaemia protein FANCD2, the main checkpoint kinase during replication stress ATR, the RAD51 recombinase, the BLM helicase, the DNA structure-specific endonuclease, MUS81, and the non-catalytic subunit of the XPF endonuclease, ERCC1 [20,30,51–55]. For background reading on DNA damage response proteins, we refer readers to the following reviews [56,57]. Among these proteins, FANCD2 is frequently used as a surrogate marker of the location of CFSs in human cell nuclei [20]. Why this protein localizes to CFSs in this way is still debated, but one possible explanation is that it associates with R-loops generated at CFSs and elsewhere in the genome [58–61]. For further details on the proteins required to promote CFS stability, we refer readers to the following articles [31,62].

### 2.3. Telomeres

Telomeres are the specialized nucleoprotein structures that protect the natural ends of linear eukaryotic chromosomes from being recognized as DSBs. Because of this, the chromosome end is prevented from triggering either a DNA damage response or a chromosome end-to-end fusion [63]. Mammalian telomeres are composed of TTAGGG repeats bound by a six-protein complex called shelterin. The G-rich 3′ terminating strand forms a ssDNA overhang that is also necessary for telomere maintenance because it can invade into the double-stranded telomeric DNA to form a protective structure called the t-loop [64,65]. The shelterin complex is composed of dsDNA-associated factors, TRF1 and TRF2, and the ssDNA overhang-binding protein, POT1, together with the TIN2, TPP1 and RAP1 proteins (reviewed in [66,67]). Despite their constitutive heterochromatic nature, telomeres can be transcribed by RNA polymerase II into a long noncoding RNA called TERRA [68].

The unique structural and functional features of telomeres promote chromosome stability. However, these features also create challenges for the DNA replication machinery, and telomeres are intrinsically difficult to replicate. For example, replication fork progression can be impeded by one or more of the following: (i) G-rich repetitive sequences that form DNA secondary structures such as G-quadruplexes; (ii) the tightly bound shelterin complex, which can form a physical blockade to the replisome; (iii) the t-loop, which can inhibit replication fork progression if not appropriately dismantled and (iv) the formation of TERRA-associated R-loops. Another feature of telomeric replication is that it is unidirectional. This might contribute to the difficult-to-replicate nature of each telomere because there are no available ‘downstream’ replication origins that can be activated in the event of prolonged or irreparable replication fork stalling [69,70].

Telomeres have been shown to phenotypically resemble fragile sites, in that they exhibit overt fragility under replication stress conditions [71]. Because telomeric fragility is technically challenging to detect, the use of telomeric fluorescence *in situ* hybridization-based staining is widespread. Fragility at telomeres is generally defined as the presence of either multi-telomeric signals or elongated telomeres. Similar to CFSs, low dose aphidicolin treatment induces telomere fragility. A number of factors suppress this fragility, including telomere-associated proteins, such as TRF1, as well as two DNA helicases, BLM and RTEL1, which are recruited to telomeres during S-phase [71–74]. Two recent reviews provide a more comprehensive discussion of the key proteins required for telomere replication and stability [69,75].

Owing to the requirement for DNA replication to begin from an RNA primer, it is not possible to fully replicate the lagging strand template at the very end of a chromosome (known as the ‘end replication problem’). As a consequence, telomeres shorten with each round of DNA replication in somatic cells. In the absence of telomere maintenance mechanisms, cells can undergo a limited number of divisions before they arrest in a state termed replicative senescence [76,77]. To avoid this fate, stem cells and germ cells use the telomerase reverse transcriptase enzyme, which carries its own RNA as a template for telomere extension [78–80]. Cancer cells also reactivate telomere maintenance mechanisms to enable replicative immortality [1]. Around 90% of human cancers activate expression of telomerase [81], while the remaining 10% use a process called ALT (the alternative lengthening of telomeres). ALT appears to be more prevalent in those rare tumours of mesenchymal origin, rather than the more common epithelial cancers. ALT is a homologous recombination-mediated telomere maintenance pathway [82–84]. The phenotypes of ALT cells are the absence of telomerase, a heterogeneous telomere length, the presence of a specialized PML body composed of DNA damage and repair proteins at telomeres (ALT-associated PML bodies; APBs), an increased frequency of telomere sister chromatid exchanges, and the presence of extra-chromosomal telomeric DNA [85,86]. In addition, ALT cells frequently exhibit loss of the ATRX protein and increased expression of TERRA RNA [87–89]. ALT telomeres also appear to be sensitive to DNA replication stress, as evidenced by an increased propensity to exhibit fragility. This might be due to an elevated level of TERRA transcription [90–92], as ALT cells are thought to be more permissive for transcription due to an altered chromatin compaction [87]. Consistent with this, the depletion of the two paralogues of the histone chaperone ASF1 (ASF1a and ASF1b) induces ALT phenotypes, including increased APBs and C-circles. Therefore, enhanced replication fork stalling caused by dysfunctional histone dynamics might trigger the induction of ALT at telomeres [93].

An analogous mechanism to ALT is conserved in lower eukaryotes. In the absence of telomerase in yeast, the rare accumulation of so-called Type I and Type II survivors is driven by the use of a homologous recombination-based mechanism for telomere maintenance. The precise mechanism by which these survivors arise is still not clear, but a recombination-driven process called break-induced replication (BIR) is implicated in ALT in yeast, which will be discussed further in the MiDAS section below [94]. Recent studies identified two possible mechanisms for the ALT process in human cells. When TRF1 was fused to a bacterial endonuclease FokI (TRF1-FokI) in order to induce a DSB specifically at telomeres, the resultant critically short or dysfunctional telomeres were ‘healed’ using either of two recombination-based telomere maintenance pathways [95,96]. One of these putative ALT mechanisms depends upon the major recombinase protein RAD51, but the other does not. The RAD51-dependent process, which requires a conventional homology search, apparently uses the HOP2–MND1 heterodimer involved in meiotic recombination [95]. By contrast, the RAD51-independent process utilizes a pathway that was termed ‘break-induced telomere synthesis'. This process occurs outside of S-phase and requires POLD3, RFC1 and PCNA, but not HOP2-MND1 [96].

## 3. Mitotic DNA synthesis

Although the bulk of DNA replication is completed during S-phase, it has been known for some time that certain regions of the genome can show a delay in completion of DNA replication. While this was generally assumed to be occurring during the G2 phase, recent data indicate that DNA synthesis can still occur after the cells have initiated the prophase of mitosis. In this section, we review the evidence that a form of MiDAS occurs at CFSs and telomeres.

### 3.1. MiDAS at common fragile sites

The delayed replication of CFSs following DNA replication stress was first reported almost two decades ago [33]. Indeed, many studies have demonstrated that CFS replication can sometimes occur outside of a conventional S-phase [51,97,98]. Recently, our laboratory demonstrated that DNA synthesis at CFSs could be detected even after cells had entered the prophase of mitosis [16]. Following the initiation of chromosome condensation and the activation of the prophase pathway to eliminate sister chromatid cohesion from chromosome arms, any remaining under-replicated CFS loci trigger a non-canonical mode of mitotic DNA synthesis (which we termed ‘MiDAS’) [16]. Although this process is detectable in all cell types, it is particularly prevalent in transformed cancer cell lines that exhibit aneuploidy [16,99].

**Figure 1.**
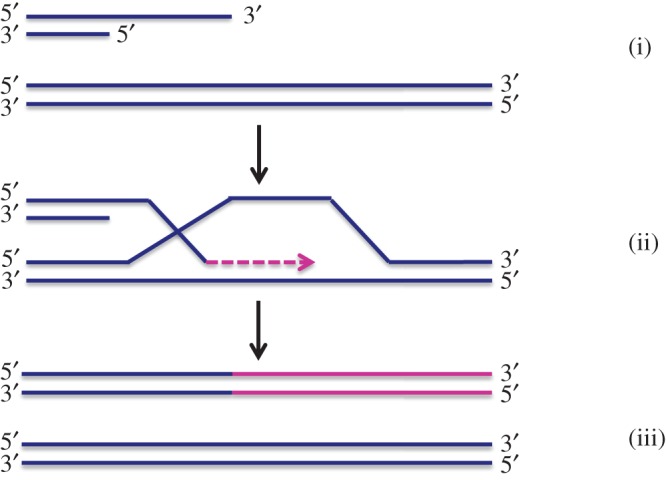
Key steps involved in the BIR pathway. BIR initiates from a double strand DNA end that has been resected to generate a 3′ single stranded DNA overhang (i). This overhang then invades into a homologous DNA duplex to form D-loop (ii) followed by DNA synthesis and D-loop migration and subsequent initiation of complementary strand synthesis (iii) [103].

The MiDAS pathway differs from conventional DNA replication in that it frequently uses a conservative form of DNA synthesis. This was revealed by the distinctive patterns of nascent DNA (labelled with EdU) on mitotic chromosomes [100]. In this respect, MiDAS resembles BIR in yeast, which uses a conservative form of DNA synthesis [101,102] to repair one-ended DSBs, such as those arising at broken/collapsed replication forks. BIR may also be used to maintain ALT telomeres, as discussed above. BIR involves the invasion of the 3′ single-stranded DNA overhang derived from a resected DSB into a homologous double-stranded DNA, to form a D-loop that allows the invading DNA to prime new DNA synthesis (figure 1; reviewed in [103–105]). The first evidence for BIR at difficult-to-replicate loci in human cells came from analysis of oncogene-induced DNA replication stress. A POLD3-dependent form of BIR was proposed as the pathway of choice for the repair of the collapsed replication forks following cyclin E overexpression [106]. Consistent with this, BIR in yeast requires Pol32, the yeast homologue of POLD3 [107].

**Figure 2.**
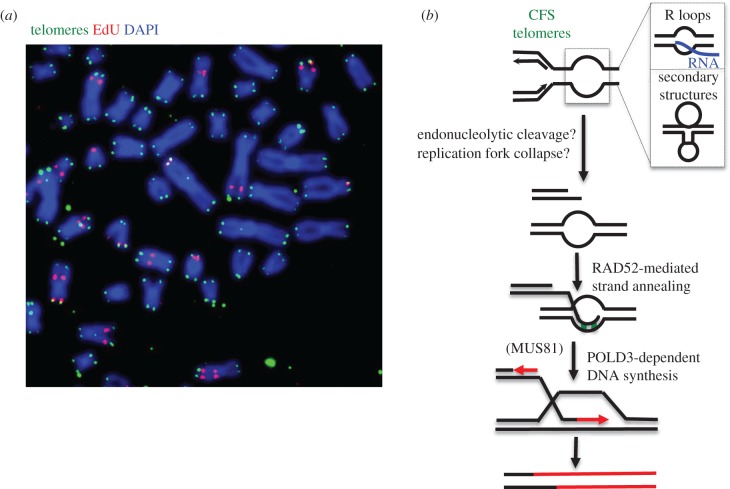
MiDAS at difficult-to-replicate loci. (*a*) Representative image of the detection of MiDAS in HeLa cells treated with low dose aphidicolin (APH). The ongoing DNA synthesis marked by EdU incorporation (red) can be seen in relation to the telomeric DNA ends (green). DNA is stained with DAPI (blue). (*b*) A current model for MiDAS [100]. The cartoon shows how a BIR-like process (figure 1) might occur at telomeres and CFSs when a stalled replication fork is broken and new DNA synthesis is activated (red). The fact that the process is RAD51-independent (which is unusual for BIR) suggests that perhaps the annealing of the broken arm of the fork occurs at a DNA structure that is already in an open conformation due to the presence of an R-loop or a DNA secondary structure.

In addition to POLD3, MiDAS at CFSs in humans requires the SLX4 scaffold protein, the MUS81-EME1 endonuclease, and RAD52, but is RAD51-independent. Rather, the depletion of RAD51 causes an increase in MiDAS, suggesting that MiDAS is upregulated in the absence of RAD51. The requirement for RAD52, but not RAD51, is intriguing given that most BIR in yeast requires Rad51, and the initiation of BIR is believed to require a DNA strand invasion event that requires Rad51. If MiDAS does occur via a BIR-like pathway, then this suggests that, under specific circumstances, RAD52 can catalyse an analogous reaction to strand invasion that permits DNA synthesis to be primed. For example, MiDAS may be a microhomology-mediated form of BIR (figure 2) [16,100]. Further reading on microhomology-mediated BIR can be found in the following articles [104,105,108–110].

### 3.2. Mitotic DNA synthesis at telomeres

In addition to CFSs, events analogous to MiDAS have also been observed at telomeres [91,99,111]. This telomeric MiDAS has been characterized mainly in conjunction with the ALT mechanism. For example, in a recent study, telomeric DSBs in ALT cells were shown to be repaired by a conservative, POLD3-dependent, form of telomere DNA synthesis. Moreover, this repair process did not require RAD51, making it analogous to the MiDAS pathway at CFSs [96,112]. Surprisingly, however, this process was not specific for cells using the ALT mechanism, but was operational in all cell lines in which a site-specific telomeric DSB was generated [96]. Hence, this form of so-called ‘break-induced telomere synthesis' might be a process that occurs only in the context of the formation of a DSB within a telomere. Nevertheless, break-induced telomere synthesis is proposed to be analogous to BIR in yeast cells that show an ALT-like telomere maintenance system [113]. Indeed, recent work from the Shay group identified a RAD51- and BRCA2-independent, but RAD52-dependent, MiDAS pathway operating at ALT telomeres, which bears many of the hallmarks of BIR and CFS MiDAS [91]. In another recent study, the loss of the Pol*η* translesion DNA polymerase was shown to induce telomeric MiDAS in ALT cells [111]. Pol*η* has been demonstrated previously to process stalled replication forks at CFSs in S-phase in order to counteract fragile site instability [97]. This shared role for Pol*η* at CFSs and telomeres likely reveals a general mechanism used by cells to counteract DNA replication stress at difficult-to-replicate loci. Finally, there are contradictory findings regarding the requirement for ATR in telomeric MiDAS. The Shay group has shown that telomeric MiDAS is ATR-dependent, while our group found that ATR inhibition exacerbates telomeric MiDAS in a manner similar to that seen with CFS-MiDAS [91,99]. The reason for this difference is not known and requires further investigation. What is generally agreed is that the telomeric MiDAS pathway uses a largely conservative form of DNA synthesis that is analogous to BIR in yeast. Telomeric MiDAS shares many features with CFS-MiDAS, including a requirement for SLX4, RAD52 and POLD3, but differs form CFS-MiDAS in being independent of MUS81 (figure 2) [91,96,99].

In contrast to some of the studies reviewed above, our characterization of the telomeric MiDAS pathway has shown that telomeric MiDAS can be detected in most cell lines exposed to aphidicolin, irrespective of their ALT status. We also observed that the cells with the highest levels of basal telomeric MiDAS are those with the longest telomeres, particularly if they use the ALT pathway. Nevertheless, we also revealed a significant level of basal MiDAS in telomerase-positive cells that display a high degree of aneuploidy. Indeed, although aphidicolin can activate telomeric MiDAS, the presence of aneuploidy seems to be a key factor in determining whether the telomeric MiDAS pathway is used in cells exposed to DNA replication stress [99]. Our data indicate that telomeric MiDAS is not synonymous with the ALT mechanism. Furthermore, it is known that several independent means of inducing DNA replication stress, such as the overexpression of cyclin E, treatment of cells with a G-quadruplex stabilizing ligand (pyridostatin), and the depletion of RNase H1 to increase R-loop formation, all increase telomeric MiDAS levels [5,6,91,114,115]. Hence, we propose that MiDAS is a general mechanism for counteracting DNA replication stress at any form of difficult-to-replicate region of the genome, including CFSs and telomeres. Further work will be required to assess whether other difficult-to-replicate loci such as the rDNA also depend on MiDAS for their stability following induction of DNA replication stress.

ALT cells appear to exhibit higher levels of DNA replication stress and telomeric fragility than telomerase-positive cells [90–92]. An increase in the frequency of replication–transcription collisions (generating R-loops) and/or the possibility that the longer telomeres in ALT cells are more prone to form replisome-blocking G-quadruplexes might explain the higher basal levels of telomeric MiDAS observed in ALT cell lines. One speculative mechanism that ALT cells might use to minimize telomere instability is to co-opt the TERRA RNA for activating DNA synthesis. Consistent with this, TERRA-dependent R-loops, rather than being pathological, might promote telomere maintenance in ALT cells, as evidenced by telomere shortening upon RNase H1 overexpression [90].

## 4. What happens when MiDAS fails?

**Figure 3.**
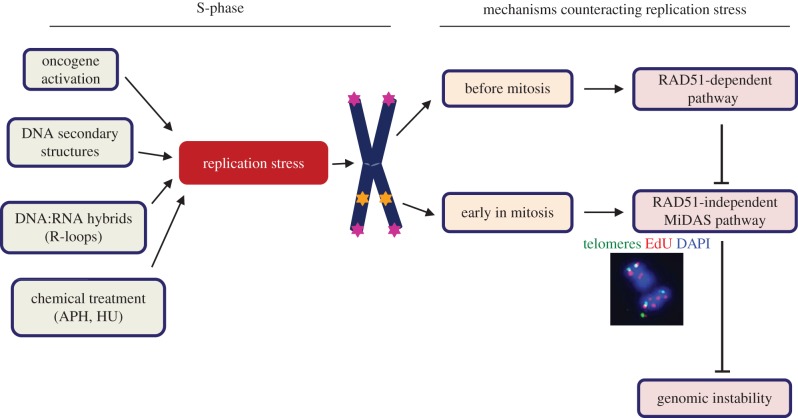
The mechanisms for counteracting replication stress that prevent genome instability. Replication stress at difficult-to-replicate loci such as CFSs and telomeres (indicated by stars on the chromosome arms) is initially dealt with via a canonical RAD51-dependent pathway before the cells enter into mitosis. When this pathway fails, a non-canonical, RAD51-independent, process (MiDAS) takes over to prevent genomic instability. APH, aphidicolin, HU, hydroxyurea.

**Figure 4.**
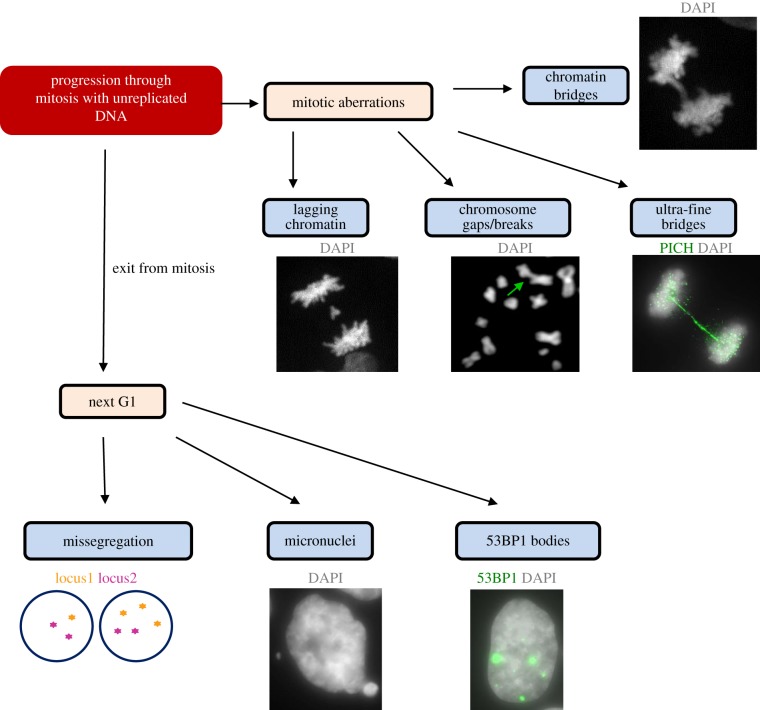
What happens when MiDAS fails? The consequences of MiDAS failure and progression through mitosis with unreplicated DNA could be not only the formation of mitotic aberrations such as anaphase bridges, lagging chromatin and chromosome breaks/gaps, but also genomic instability in the next G1 cell cycle of daughter cells. Mitotic anaphase bridges, which are classified as either chromatin bridges or ultra-fine bridges, are observed when cells attempt to segregate incompletely replicated or unresolved DNA structures. This can lead to the daughter cells acquiring an incorrect chromosome number/structure, to the formation of micronuclei, and to the formation of so-called 53BP1 nuclear bodies in the daughter G1 cells.

Our contention is that, in order to counteract replication stress, transformed/cancer cells use a RAD51-dependent homologous recombination repair pathway in late S/G2 to try to effect the completion of replication. If this fails for any reason, and cells enter mitosis with unreplicated DNA, they then switch to the RAD52-dependent MiDAS pathway. MiDAS at both CFSs and telomeres seems to constitute a final attempt (analogous to a salvage pathway) to complete DNA synthesis and hence to prevent extensive genomic instability (figure 3). Indeed, under-replicated regions, or unresolved DNA structures, can lead to mitotic aberrations such as chromatin bridges, UFBs, lagging chromatin and chromosome gaps and breaks. Furthermore, they can also generate micronuclei and 53BP1 bodies in the subsequent G1 phase (figure 4) [116–119]. MiDAS is, therefore, important for suppressing these abnormalities by counteracting the high levels of DNA replication stress arising at difficult-to-replicate loci. Consistent with this, depletion of key MiDAS factors (e.g. RAD52), or acute inhibition of MiDAS by exposure to a high dose of aphidicolin in mitosis, have been shown to directly induce mitotic aberrations and chromosome missegregation [16,100].

## 5. MiDAS inhibitors for cancer therapy?

The elevated levels of MiDAS observed in cancer cells make this pathway a feasible target for anti-cancer therapy [16,99]. Indeed, a reliance on MiDAS might allow cancer cells to better tolerate the chronic DNA replication stress associated with oncogene activation. Identifying the key players of MiDAS (table 1) will enable the development of strategies to target this pathway [16,51,91,96–100,111,112,120]. Potentiation of DNA replication stress to toxic levels by inhibiting the ATR kinase is a strategy being used to target the chronic DNA replication stress phenotype of cancer cells, and this would be expected to make ATR-deficient cancer cells even more reliant on MiDAS for survival. In this scenario, we propose that ATR inhibitors and MiDAS inhibitors could be an effective combinatorial strategy to trigger irreparable DNA replication stress in cells with activated oncogenes. Indeed, as a proof-of-principle, we have demonstrated that RAD52 inhibition potentiates the effects of an ATR inhibitor in cancer cells [100].

**Table 1.** The list of proteins implicated thus far in MiDAS, by either promoting or suppressing MiDAS at CFS and/or telomeres. SCEs, sister chromatid exchanges; TIFs, telomere induced foci.

proteins	implicated role in MiDAS	consequences in the absence of the protein	references
ERCC1	localized to EdU incorporation in G2/M at CFSs	increased chromatin bridges, chromosome segregation failures, mitotic catastropy, CFS fragility/expression, 53BP1 bodies	[51]
SLX4	required for CFS- and telomeric MiDAS	decreased CFS expression, increased UFBs, chromatin bridges, 53BP1 bodies	[16,99]
MUS81	required for CFS-MiDAS, but not for telomeric MiDAS	increased chromatin bridges, chromosome segregation failures, mitotic catastropy, 53BP1 bodies, decreased CFS expression	[16,51,99]
EME1	required for CFS-MiDAS	not addressed	[16]
WAPL	required for CFS- and telomeric MiDAS	decreased CFS expression, increased UFBs, chromatin bridges, 53BP1 bodies	[16]
SMC2	required for CFS- and telomeric MiDAS	decreased CFS expression, increased UFBs, chromatin bridges, 53BP1 bodies	[16]
replicative polymerases	required for CFS- and telomeric MiDAS	increased 53BP1 bodies, non-disjunction, binucleation	[16,99]
POLD3	required for CFS-MiDAS and break-induced telomere synthesis	decreased CFS expression, increased 53BP1 bodies, UFBs, decreased telomere length, increased TIFs, decreased c-circles	[16,96,112]
PLK	required for CFS-MiDAS	not addressed	[16]
TOPBP1	required for MiDAS at CFSs	increased 53BP1 bodies, binucleation	[98]
RAD52	required for CFS- and telomeric MiDAS	increased 53BP1, chromatin bridges, UFBs, micronuclei, decreased CFS expression	[91,99,100]
PCNA	required for break-induced telomere synthesis	not addressed	[96]
RFC	required for break-induced telomere synthesis	not addressed	[96]
Smc5/6 complex	required for telomeric MiDAS	decreased telomere clustering	[91]
RECQ5	required for CFS-MiDAS	decreased CFS expression, increased chromatin bridges, UFBs, micronuclei, 53BP1 bodies	[120]
MRE11	required for telomeric MiDAS	not addressed	[91]
Pol *η*	suppresses G2/M DNA synthesis at CFSs and telomeric MiDAS (not a mitosis specific protocol)	increased 53BP1 bodies, SCEs at CFSs and increased APBs, c-circles, t-SCEs, telomere fragility	[97,111]
ATR	suppresses CFS-MiDAS, contradicting reports on telomeric MiDAS	increased CFS expression	[91,96,99,100]
RAD51	suppresses CFS- and telomeric MiDAS	increased telomere fragility, increased TIFs	[91,96,100]
BRCA2	suppresses CFS- and telomeric MiDAS	increased telomere fragility, increased TIFs	[91,100]
HOP2	suppresses telomeric MiDAS	not addressed	[96]
TIMELESS/TIPIN complex	suppresses telomeric MiDAS	increased telomere clustering	[91]

## 6. Concluding remarks

Recent studies have expanded our understanding of the MiDAS pathway and the consequences of DNA replication stress at difficult-to-replicate loci. However, several questions still remain unanswered. For example, the predominant MiDAS pattern whereby EdU incorporation is seen on only one sister chromatid at CFSs and telomeres seems to be a signature of the conservative BIR pathway [91,99,100,112]. However, EdU incorporation also occurs on both sister chromatids in 20%–40% of the cases, and sometimes shows variegated signals. It is possible, therefore, that different sub-pathways of MiDAS are deployed at different types of DNA structures. For example, fork cleavage may create a one-ended DSB for BIR, whereas under-replicated regions may be unwound to generate two ssDNA gaps that are repaired via other means. To better understand these processes, it will be of interest to identify any conditions that alter the pattern of EdU incorporation in mitosis. Furthermore, the molecular mechanisms of MiDAS could be characterized further through the use of site-specific DNA replication barriers which could be used to create defined regions of the genome that are maintained exclusively by MiDAS.

The current model of MiDAS is analogous to the RAD51-independent BIR mechanism in yeast [100]. Nevertheless, it appears that RAD51-dependent repair occurs during the late S/G2 phases in an attempt to complete DNA synthesis before mitosis, implying that RAD51-independent MiDAS only occurs in mitosis as a back-up ‘salvage’ pathway [95,96,100]. It will be interesting to test how both types (RAD51-dependent or independent) of BIR cooperate in human cells to repair stalled replication forks at difficult-to-replicate loci, and how their relative usage is regulated by the level of DNA replication stress and the stage of the cell cycle.

One critical deficiency in our knowledge concerns the nature of the lesion(s) that cause replication fork stalling at CFSs and telomeres in the first place. Indeed, perhaps there is no single form of ‘roadblock’ implicated in this, and therefore it does not matter how the replisome is disrupted because the end result is always the same; the replication fork will need to be rescued. Recent work has provided some evidence for this contention, in that the stabilization of either G-quadruplexes or R-loops has been shown to increase the dependence on telomeric MiDAS [91]. Finally, further work is required to improve our understanding of the putative role of RNA species (TERRA or R-loops) in MiDAS, and to determine their physiological (as well as potentially pathological) roles at these regions [121,122]. It is intriguing that various types of RNA species appear to play prominent roles that likely determine CFS and telomere stability. Of note, it remains unknown as to why many of the transcripts generated at CFSs are so large. Although R-loops are conventionally considered as pathological, increasing evidence suggests that these RNA : DNA hybrids might have important physiological roles under some circumstances.

## Data accessibility

This article has no additional data.
